# Inter-population variability of DEFA3 gene absence: correlation with haplotype structure and population variability

**DOI:** 10.1186/1471-2164-8-14

**Published:** 2007-01-10

**Authors:** Ester Ballana, Juan Ramón González, Nina Bosch, Xavier Estivill

**Affiliations:** 1 Genes and Disease Program, Center for Genomic Regulation (CRG), Barcelona, Catalonia, Spain; 2 CeGen, Spanish Nacional Genotyping Center, Barcelona, Catalonia, Spain; 3 Universitat Pompeu Fabra (UPF), Barcelona, Catalonia, Spain

## Abstract

**Background:**

Copy number variants (CNVs) account for a significant proportion of normal phenotypic variation and may have an important role in human pathological variation. The α-defensin cluster on human chromosome 8p23.1 is one of the better-characterized CNVs, in which high copy number variability affecting the *DEFA1 *and *DEFA3 *genes has been reported. Moreover, the *DEFA3 *gene has been found to be absent in a significant proportion of control population subjects. CNVs involving immune genes, such as α-defensins, are possibly contributing to innate immunity differences observed between individuals and influence predisposition and susceptibility to disease.

**Results:**

We have tested the *DEFA3 *absence in 697 samples from different human populations. The proportion of subjects lacking *DEFA3 *has been found to vary from 10% to 37%, depending on the population tested, suggesting differences in innate immune function between populations. Absence of *DEFA3 *was correlated with the region's haplotype block structure. African samples showed a higher intra-populational variability together with the highest proportion of subjects without *DEFA3 *(37%). Association analysis of *DEFA3 *absence with 136 SNPs from a 100-kb region identified a conserved haplotype in the Caucasian population, extending for the whole region.

**Conclusion:**

Complexity and variability are essential genomic features of the α-defensin cluster at the 8p23.1 region. The identification of population differences in subjects lacking the *DEFA3 *gene may be suggestive of population-specific selective pressures with potential impact on human health.

## Background

Defensin genes encode a family of small cationic peptides that act as antimicrobial mediators of the innate immune system [1]. Defensins are arginine-rich peptides and invariably contain disulfide-linked cysteine residues, whose positions are conserved [2]. The two main defensin subfamilies, α- and β-defensins, differ in the length of the peptide segments between cysteine residues and in the arrangement of disulphide bonds that link them. β-defensins have been found in most vertebrate species, whereas α-defensins are specific to mammals [3]. Based on their adjacent chromosomal location, similar precursor peptides and gene structures, it has been postulated that all vertebrate defensins arose from a common gene precursor [4]. While the efficacy of individual defensins against specific infectious agents varies, they have shown antimicrobial activity against gram-negative and gram-positive bacteria, fungi and enveloped viruses [1,5]. At high concentrations, some defensins are also cytotoxic to mammalian cells, as cells exposed to high amounts of defensins in inflamed tissues generate pro-inflammatory signals that can contribute to tissue injury [1]. In humans, most of the genes encoding α- and β-defensins are located in clusters on chromosome 8p23.1 [6,7]. Within the region, two different defensin clusters can be distinguished: a telomeric cluster mostly containing α-defensin genes (*DEFB1*, *DEFA6*, *DEFA4, DEFA1, DEFT1, DEFA3 *and *DEFA5*) and at least two centromeric clusters of β-defensin genes (*DEFB109p*, *DEFB108*, *DEFB4*, *DEFB103*, *DEFB104*, *DEFB106*, *DEFB105 *and *DEFB107*) [7].

Chromosome band 8p23.1 is known to be a frequent site of chromosomal rearrangements mediated by low copy repeats (LCRs) or segmental duplications (SDs). It has been described that as many as one in four individuals from the general population carry a 4.7 Megabase (Mb) inversion of the region [8-10]. In addition, copy number variability involving both α-defensin (*DEFA1 *and *DEFA3*) and β-defensin (*DEFB4*, *DEFB103 *and *DEFB104) *genes in chromosome 8p23.1 has been well detected and characterized [11-14]. The number of *DEFA1 *and *DEFA3 *gene copies has been reported to range from 4 to 11 in a sample of 111 subjects, the *DEFA3 *allele being completely absent in 10% of them [12]. Gene nomenclature for *DEFA1*, *DEFT1 *and *DEFA3 *has been replaced by *DEFA1A3*, following recommendations of Aldred et al, since these genes have been considered as being part of a copy number variant (CNV) region [14]. In another study, Linzmeier and colleagues determined copy numbers of the *DEFA1 *and *DEFA3 *alleles in 27 subjects and found between 5 and 14 copies per diploid genome, with *DEFA3 *being absent in 26% of them [14].

Despite *DEFA1 *and *DEFA3 *being considered as members of the same CNV *(DEFA1A3)*, they encode different peptides, HNP-1 and HNP-3, respectively. The mature HNP-1 and HNP-3 peptides differ only in their N-terminal amino acid, due to a single nucleotide difference, C3400A, between the *DEFA1 *and the *DEFA3 *genes [15]. This C3400A is a paralogous sequence variant (PSV) that allows discrimination between the two gene copies. The HNP-2 peptide is identical to the last 29 amino acids of both the HNP-1 and the HNP-3 peptides. HNP-2 is presumably produced from proHNP-1 and/or proHNP-3 by post-translational proteolytic cleavage [1]. It is likely that one or both genes, or another member of the *DEFA1A3 *CNV cluster encode the HNP-2 peptide. The three peptides are constitutively produced by neutrophil cell precursors and packaged in granules before mature neutrophils are released into the blood. During phagocytosis, the defensin-containing granules fuse to phagocytic vacuoles where defensins act as antimicrobial agents [15].

Recent work has shown that CNVs are a major source of genetic variation [16]. Individual variability in resistance to infectious diseases has been extensively reported [17]. However, the causes of this diversity in immune function are poorly understood. CNVs involving immune genes could contribute to the differences in innate immunity between individuals and influence predisposition and susceptibility to diseases, as it has been shown for human immunodeficiency virus and AIDS [18]. Thus, it is important to analyze the impact of defensin gene CNVs on human health, both in healthy volunteers and in patients with disease [1,19]. In this report we have studied the presence of *DEFA3 *in samples from different human populations. For this purpose, we used the International Haplotype Map (HapMap) Project collection and a cohort of Spanish healthy individuals.

## Results

### Differences in the proportion of *DEFA3 *absence between populations

We have analyzed 786 samples from four populations with ancestry in Europe, Africa or Asia (the HapMap collection), including Spanish healthy individuals. The source used for this study was the HapMap collection of 269 samples utilized by the International HapMap Consortium for the study of human genomic variation, initially through the investigation of SNPs and their associated haplotypes [20], and 180 additional HapMap samples. This collection comprises four populations: 30 parent-offspring trios (90 individuals) of the Yoruba from Ibadan, Nigeria (YRI), 30 parent-offspring trios (90 individuals) of European descent from Utah, USA (CEU), 45 unrelated Japanese from Tokyo, Japan (JPT) and 44 unrelated Han Chinese from Beijing, China (CHB). In addition, 30 Yoruban trios, 45 unrelated Japanese and 45 unrelated Chinese from the HapMap collection, but not genotyped in the HapMap project, were analyzed. The Spanish samples were 336 unrelated blood donor controls, all of Caucasian origin. Genomic DNA from EBV-transformed lymphoblastoid cell-lines was used. As Chinese and Japanese allele frequencies are found to be very similar [20], the analysis was performed combining both datasets, resulting in four different groups of samples tested: two Caucasian groups (CEU and Spanish general population subjects), Yoruba and Chinese/Japanese.

The coding sequence of *DEFA1 *and *DEFA3 *differs only by a single nucleotide (C3400A), which allows distinguishing between *DEFA1 *and *DEFA3 *by *Hae*III digestion, since a restriction site for this enzyme is absent in the *DEFA3 *sequence. All samples had at least one *DEFA1 *copy, but *DEFA3 *was absent in several subjects of all populations. *DEFA3 *was absent in different proportions depending on the population tested, ranging from 10% in the Chinese/Japanese dataset to 37% in the Yoruba samples (Table 1). There were statistically significant differences for the absence of *DEFA3 *when comparing Yoruba samples with each of the other population groups (Table 1) or with the total of non-Yoruban unrelated subjects (p < 0.001). As both Caucasian and Yoruba samples are trios, inheritance of the *DEFA3 *allele could also be assessed, showing no abnormal segregation in any of the trios analyzed (data not shown).

### Segmental duplications and genomic organization of α-defensin cluster

**Figure 1:**
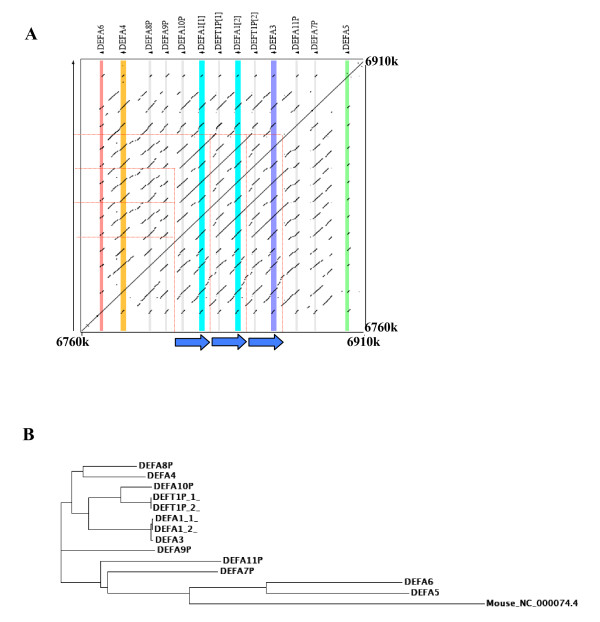
Genomic organization of α-defensin cluster at 8p23.1 region. **A**. Dot-plot of the PipMaker alignment of the 150 kb region containing the α-defensin cluster. The high density of segments showing alignment is due to the presence of defensin genes and pseudogenes, sharing a common genomic structure. Vertical coloured lines represent α-defensin genes and grey lines correspond to pseudogenes localizations. The 19 kb duplicons are indicated by arrows. Note that all human defensins are transcribed from the same direction. **B**. Phylogenetic tree of human α-defensins. Mouse ortholog of human *DEFA1 *gene was included as a root.

The genomic organization of the α-defensin cluster was precisely defined by PipMaker analysis [21]. For this analysis, a region of 150 kb containing the whole α-defensin cluster on 8p23.1 was used (based on May 2004 human genome assembly). The alignment of the region against itself identified different sequences with high homology, which correspond to six α-defensin genes (*DEFA6*, *DEFA4, DEFA1*, *DEFA3 *and *DEFA5*), six α-defensin pseudogenes (*DEFA8P*, *DEFA9P*, *DEFA10P*, *DEFA11P *and *DEFA7P*) and one θ-defensin pseudogene (*DEFT1P*) (Figure 1a). Such clustered organization of α-defensin genes is common in other species, suggesting that α-defensin have arisen from a common ancestor by gene duplication followed by diversification [3]. Phylogenetic analysis of all human α-defensin genes and pseudogenes showed that *DEFA5 *and *DEFA6 *seem to be the ancestral genes. All pseudogenes are clustered together with these two genes, with the exception of *DEFA10P *and *DEFT1P*, which are closely related with *DEFA1 *and *DEFA3 *(Figure 1b).

**Figure 2:**
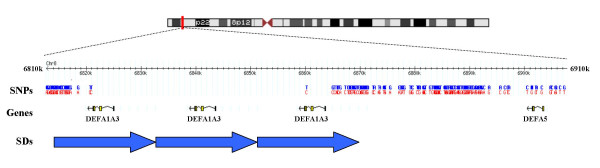
Schematic representation of a 100-kb region of human chromosome 8p23.1 containing the *DEFA1 *and *DEFA3 *genes. Gene and SNP positions are based on May 2004 genome assembly (hg17), in which three copies of the *DEFA1A3 *are annotated. The non-homogeneous distribution of SNPs at the telomeric and centromeric regions of the *DEFA1A3 *cluster is clearly seen.

Three copies of a 19-kb repeat unit were identified within the α-defensin cluster, which correspond to the *DEFA1A3 *CNV, previously reported to be variable in copy number between individuals (Figure 2) [12,14]. Each of the 19-kb repeats contained a copy of the *DEFA1 *or *DEFA3 *genes, together with a pseudogene, either *DEFA10P *or *DEFT1*. *DEFA10P *and *DEFT1P *have a high sequence identity and are closely related in the phylogenetic analysis, which is in accordance with the theory that primate specific θ-defensins evolved from α-defensins after divergence of the primates from other mammalian species [3] (Figure 1b). Variation in both number and position of *DEFA1 *and *DEFA3 *alleles has been reported, indicating that these genes are located in interchangeable variant cassettes within tandem gene arrays [12,14]. Thus, the existing diversity in *DEFA1/DEFA3 *copy number and localization is probably the result of unequal crossing-over events between tandem arrays [12]. Interestingly, multiple copies of *DEFA1*, but not the *DEFA3 *gene, can be *in silico *identified in chimpanzee by BLAST sequence similarity searches. On the other hand, in the case of Rhesus macaque the *DEFA5 *gene is present in multiple copies, suggesting a different evolutionary pattern driven by the responses to specific microbial challenges [3].

HapMap samples have been tested for the presence of CNVs by two different techniques Affymetrix SNP array and BAC array [22]. *DEFA1A3 *region was identified as a CNV in 23 subjects (3 Caucasian, 7 Yoruban, and 13 Chinese/Japanese), but only in four cases where a gain or loss was detected, *DEFA3 *is absent. Copy number variation in the *DEFA1A3 *region is reported to be much more common than the variation identified by Redon et al [22]. However, the small size of the *DEFA1A3 *CNV makes it undetectable with BAC arrays. Moreover, the presence of segmental duplications in the region entails a bad SNP coverage of the region by the Affymetrix SNP array, which does not allow an accurate detection of the CNV. Thus, the study of this CNV for association purposes has to be performed by quantitative methods or by the analysis of paralogous sequence variants.

### Patterns of linkage disequilibrium for *DEFA1A3 *in HapMap samples

A region of 100 kb, spanning from 6,810,001 bp to 6,910,000 bp, which contains the *DEFA1A3 *cluster and the single copy gene *DEFA5 *was chosen for the linkage disequilibrium analysis (based on human genome assembly hg17) (Figure 2). The HapMap data for the *DEFA1A3 *region included around 150 SNPs for each population (151 Caucasian, 169 Yoruba, 158 Japanese and 154 Chinese). However, only 136 of the SNPs had genotype data in all four populations. Interestingly, almost all genotyped SNPs are located outside the *DEFA1A3 *cluster (Figure 2). The absence of genotyped SNPs in the *DEFA1A3 *cluster is in agreement with the presence of segmental duplications that include the *DEFA1A3 *genes. Thus, the non-homogeneous distribution of SNPs within the region could be at least partially explained by the presence of high homologous repeated sequences. Genotyping errors enhanced by the presence of *DEFA1/DEFA3 *tandem gene arrays could have lead investigators to discard SNPs located within this region.

Of the 136 SNPs analyzed in all four populations, 55 were monomorphic in at least one of them (28 out of the 55 SNPs were monomorphic in all populations). Monomorphic SNPs can be used to measure genetic variability, by analyzing their distribution in the different populations. The Chinese and Japanese groups had the highest proportion of monomorphic SNPs (34%) which was very similar to that observed for Caucasian samples (31%), whereas the Yoruba samples had the smallest number of monomorphic SNPs (24%). This indicates that genetic variability is higher within Yoruba samples, while Chinese/Japanese and Caucasian populations show similar proportions of genetic variability. This higher variability for Yoruba samples is similar to that detected in the HapMap analysis for the whole genome [20]. Interestingly, the proportion of monomorphic SNPs in this region is about 10% higher for each population group than the average reported for the HapMap data [20].

**Figure 3:**
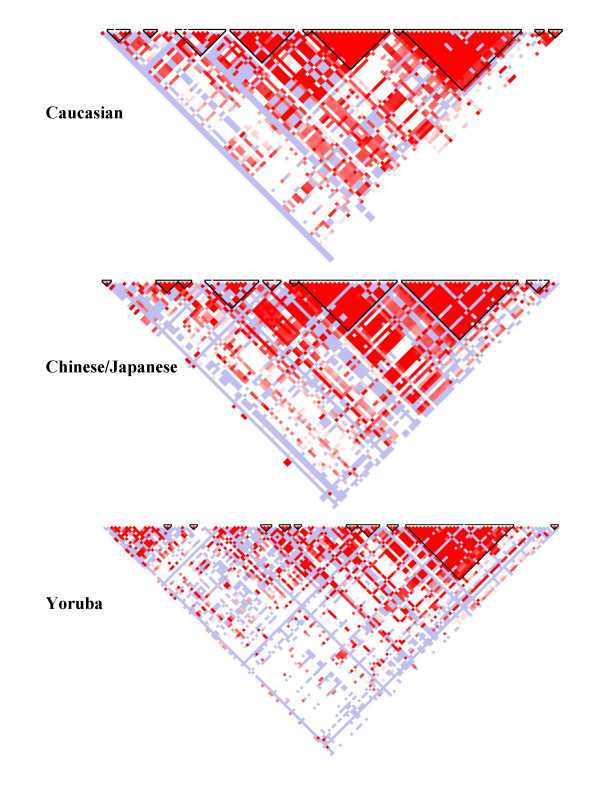
Haplotype blocks in the 100-kb region of *DEFA1A3 *cluster on human chromosome 8p23.1 generated by Haploview in Caucasian, Japanese/Chinese and Yoruba populations.

The patterns of linkage disequilibrium (LD) in each population are summarized in Figure 3. The Yoruba samples show the lowest LD, the greatest variability and smaller haploblocks compared to Caucasian or Chinese/Japanese samples, which have similar patterns of LD. The differences observed in LD patterns between populations are in accordance with *DEFA3 *locus absence results; the Yoruba samples showing highest LD variability and also having the highest proportion of *DEFA3 *absence.

### *DEFA1A3 *region haplotype association with *DEFA3 *absence in HapMap samples

**Figure 4:**
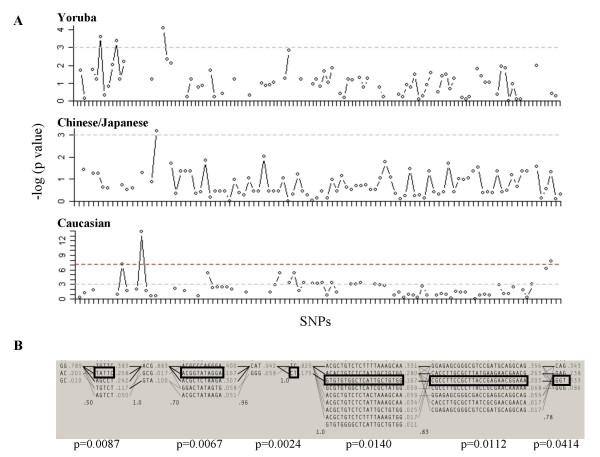
Association of SNPs in the 100-kb region of *DEFA1A3 *cluster with the absence of the *DEFA3 *gene. **A**. Diagram with the association results testing each SNP with *DEFA3 *absence under a recessive mode of inheritance. The red line indicates significance after Bonferroni correction. Positive association was only detected in Caucasian population. **B**. Haplotype blocks defined by the haploview program in the Caucasian population. The estimated frequency of each haplotype is depicted on its right hand side. The boxes represent the haplotype found associated to *DEFA3 *absence and the corresponding p-value is shown.

To assess whether *DEFA3 *is inherited together with neighbor SNPs, an association study was performed using the HapMap data for the 100-kb region including the *DEFA1A3 *cluster. All the SNPs of the region genotyped in the HapMap project were tested for association with the C3400A PSV, which defines the presence or absence of *DEFA3 *gene, respectively. No association for any of the genotyped SNPs was found in the Yoruba or Japanese/Chinese populations. However, a significant association was found between absence of *DEFA3 *and 18 SNPs in the Caucasian samples, under a recessive mode of inheritance (Figure 4a, Additional file 1). Association between estimated haplotypes within defined LD blocks and the C3400A PSV has also been tested. Again, the Caucasian group was the only one in which significant association was obtained (Figure 4b). Moreover, the associated haplotype spans nearly the whole 100-kb region, indicating a lack of recombination between the LD blocks when *DEFA3 *gene is absent. The frequency of *DEFA3 *lacking haplotype's would be similar to that estimated by the Haploview program, which varies from 16%–33% depending on the haplotype block (Figure 4b). This estimation correlates well with the observed frequency of *DEFA3 *absence in Caucasians (15%).

## Discussion

Several studies have recently reported a previously unknown high prevalence of copy number variation in humans [16]. A recent study of CNVs in the HapMap samples has defined over 1400 CNV regions [22]. On average, each individual varies at over 100 CNVs, representing about 20 Mb of genomic DNA difference. It has been suggested that CNVs account for a significant proportion of human normal phenotypic variation. It is thought that CNVs may also have an important role in the pathological variation in the human population [16,23]. Analyses of the functional attributes of currently known CNVs reveal a remarkable enrichment for genes that are relevant to molecular-environmental interactions and genes that influence response to specific environmental stimuli, such as genes involved in immune response and inflammation [16].

CNVs involving α- and β-defensin genes (*DEFA1A3 *and *DEFB4/DEFB103A) *in the 8p23.1 region have been extensively characterized [12-14]. From a pathologic point of view, it is likely that α- and/or β-defensin CNVs affect the function and effectiveness of innate immunity. Such effects could be influenced by the frequent absence of the *DEFA3 *allele. In the present work, we have tested the absence of the *DEFA3 *allele in different human populations, finding significant differences between them, which could be indicative of differences in innate immune function between populations. This is not surprising since the different human population groups have been exposed to different environments regarding infectious agents and other factors. One obvious way by which CNVs result in human phenotypic diversity is by altering the transcriptional levels of the genes which vary in copy number [16]. In addition, it has been postulated that retention of duplicate genes, rather than mutation to pseudogenes or neofunctionalization, is due to the generation of increased amounts of a beneficial product [24]. This could be the case of *DEFA1A3 *in which variation in *DEFA1 *and *DEFA3 *copy number, and *DEFA3 *absence could underlie variable resistance to infection among individuals. Different selective pressures acting in each geographic region could likely explain population differences in DEFA3 absence.

Taudien and colleagues by manual clone-by-clone alignment significantly improved the assembly of defensin 8p23.1 locus, providing *in silico *evidences of the experimentally verified variability in defensin copy number and better representing the locus diversity [7]. The exceptional genomic complexity and heterogeneity of the human 8p23.1 locus and the prominent role of defensins in the innate immunity framework raise the question of whether individual patterns of haplotypes, together with the variability in defensin genes copy number, affect the functionality of the defensin system. To address this issue, Taudien et al provided a molecular approach for the determination of individual defensin gene repertoires limited to 8p23.1 β-defensin clusters and using data from a 500 bp fragment in 4 individuals [7]. In our case, we have characterized in detail the haplotype diversity and LD structure of a 100-kb region around α-defensin locus in 269 HapMap samples. The SNP distribution of the region is characteristic of the presence of segmental duplications, which result in a low-density of SNPs selected for genotyping. As previously reported for other genomic regions [25], the Yoruba samples present a higher variability than both the Chinese/Japanese and Caucasian samples. Additionally, in the Yoruban, the haploblock structures were smaller and the extent of LD between SNPs was lower, in accordance with the out-of-Africa theory for the origins of humans. The observation that the proportion of subjects lacking the *DEFA3 *gene is greater in Yoruba samples together with the fact that *DEFA3 *is thought to be human specific [12] may be an indication of the higher amount of original genetic variation among the first humans living in Africa, which afterwards migrated to other continents. The initial migration occurred as multiple, branching events and involved many founder effects in which certain haplotypes, SNPs and alleles appear to have increased in frequency in emigrant populations owing to genetic drift and different selection pressures [25]. In this sense, we observed a diminished frequency of subjects without *DEFA3 *in Caucasian and Asian samples.

When association with *DEFA3 *absence was tested, SNPs and haplotypes in the Caucasian population were the only ones to be significant. The association observed in the Caucasian samples could be the result of strong founder effect. Founder effects and, particularly, the decrease in genetic diversity resulting from continental migrations, are associated with an increased haplotype length [25]. This is observed when comparing the haplotype block patterns of the different populations analyzed, in which the Caucasian samples set has the longest haplotype blocks. Alternatively, Aldred and colleagues demonstrated that *DEFA3 *has arisen at the 5' end repeat position and has transferred to other positions within the array through unequal recombination between alleles [12], suggesting that recombination has been active in shaping diversity in the *DEFA1A3 *locus. However, our results indicate that, at least in the Caucasian samples, there has been little recombination between chromosomes with and without *DEFA3*, as we are able to find a haplotype associated with *DEFA3 *absence extending for nearly 100-kb. Moreover, as for *DEFA3 *absence, other haplotypes are likely to be associated with other patterns of CNV polymorphisms. However, other situations cannot be rule out without analyzing large pedigrees to determine unambiguously each chromosome structure at DEFA1A3 CNV.

The impact on human health of this qualitative variation in the presence of the *DEFA3 *gene product deserves to be explored in epidemiologic studies. Different studies have described differences in the function and specificity of *DEFA1 *and *DEFA3 *gene products, HNP1 and HNP3 [1,19]. In general, HNP3 is thought to be less active than HNP1 against both gram-positive and gram-negative bacteria [26], but it is expressed at about twice the level of HNP1 [12]. On the other hand, *DEFA3 *but not *DEFA1*, has been found upregulated in patients with systemic lupus erythematosus, idiopathic thrombocytopenic purpura or rheumatoid arthritis, suggesting that *DEFA3 *upregulation might be a general feature of autoimmune diseases [27,28]. Therefore, the observed differences in *DEFA3 *absence may partially explain the different population incidences of infectious and/or autoimmune diseases in which *DEFA3 *plays an important role. Future studies are needed to establish whether patterns of *DEFA3 *absence correlate with certain population microbial exposures or different prevalence of autoimmune disorders. This could also be important in determining the exact nature of *DEFA3 *function and its specificity of action, if any, against certain antigens. Last, but not least, further studies focused on the determination of the total copy number of *DEFA1A3 *units will be crucial to build the complete picture of *DEFA1A3 *CNVs' impact on human health.

## Conclusion

Complexity and variability are essential genomic features of the α-defensin cluster at 8p23.1 region. The present work gains insight into the existent variability in human populations in this specific region. The identification of population differences in the proportion of subjects lacking the *DEFA3 *gene may be suggestive of population-specific selective pressures, which should be studied in further inter-population epidemiological studies.

## Methods

### Patients and samples

The analysis was performed on 450 HapMap samples and 336 Spanish controls. Unless otherwise noted, all samples were obtained from the Coriell Institute for Medical Research. A detailed description of HapMap populations samples can be found elsewhere [20]. Written informed consent for the Spanish controls was obtained with the approval of the Institute Review Board and Ethics Committee.

### DEFA3 determination

A PCR amplification assay followed by restriction enzyme digestion (PCR-RFLP) has been used to discriminate *DEFA1 *(GenBank accession number L12690) and *DEFA3 *(GenBank accession number L12691) genes differing by a single nucleotide. A fragment of 304 bp around C3400A SNP was PCR amplified with fluorescently labelled primers (Forward 5'-TGAGAGCAAAGGAGAATGAG-3', Reverse 5'-GCAGAATGCCCAGAGTCTTC-3') and digested with HaeIII enzyme. In order to accomplish complete digestion, we used saturating conditions (2.5 U/25 μl reaction) of the enzyme to digest a short DNA fragment containing only one cutting site. In addition, in all the runs, a *DEFA3 *negative sample was included, as a positive control of the assay. About 2 μl of digestion product was added to 10 μl HiDi formamide containing ROX500 marker (Applied Biosystems) and run on an ABI 3100 capillary system (Applied Biosystems). Peaks were analysed using Genemapper software (Applied Biosystems).

### Characterization of the segmental duplications

The UCSC Genome Browser [29] served as the main source of genomic sequence, using the human genome assembly hg17. The region analysed was a 150 kb contig from 6,760,001 bp to 6,910,000 bp of chromosome 8p23.1 (based on human genome assembly hg17). Sequences were repeat-masked and aligned against itself using PipMaker [21]. The size, orientation and structure of segmental duplications can be interpreted by using the PIP and Dot-Plot output generated by PipMaker. Multiple sequence alignments and phylogenetic tree construction were carried out by using the ClustalW program [30].

### Statistical analysis

Between groups chi-square test was performed to compare the proportion of *DEFA3 *absence in different human populations. Genotyping data from HapMap public database [31] was used to test the hypothesis of association between geneticpolymorphisms and *DEFA3 *absence using logistic regression models. Odds ratios (OR)and 95% confidence intervals (95% CI) were calculated for eachgenotype compared with the homozygous for the major allele (theallele with greater frequency among individuals lacking the *DEFA3 *allele). Analyses were initially done under a codominant inheritance model (three genotypes separated). Then, simplified models were fitted: a dominant model (heterozygous grouped with the homozygous for the minor allele), a recessive model (heterozygous grouped with the homozygous for the major allele), an overdominant model (homozygous grouped) and a log-additive model (a score was assigned counting the number of minor alleles: the homozygote for the major allele was given score 0, the heterozygote score 1, and the homozygote for the minor allele score 2). The model with lowest Akaike information criteria was the recessive one (minus twice the log likelihood of the model plus the number of variables in the model) and it was selected for an easy summary of the results. P values were derived from likelihood ratio tests, and a significance level of 5% (two sided) was used for the analyses. All these analyses were performed using the SNPassoc R package [32].

Haploblocks were constructed using Haploview program [33]. Haplotypes were reconstructed using the expectation maximization (EM) algorithm implemented in the haplo.stats R package [34]. The OR and 95% CI were estimated using a generalized linear-regression framework that incorporates haplotype phase uncertainty by inferring a probability matrix of haplotype likelihoods also implemented in haplo.stats library.

## Authors' contributions

EB carried out the genetic molecular studies, the bioinformatics work and drafted the manuscript. JRG carried out the statistical analysis. NB participated in the bioinformatics work and design of the study. XE conceived the study and participated in its design and coordination, and helped to draft the manuscript. All authors read and approved the final manuscript.

## Supplementary Material

Additional File 1 Results of the association study for the three population groups. The p-values under all inheritance modes tested are shown.
Click here for file

## Figures and Tables

**Table 1:** Absence of *DEFA3 *in Caucasian, Yoruba, Chinese/Japanese reference HapMap samples and in Spanish control samples

POPULATION*	***DEFA3***	**NO *DEFA3***	**Paired chi-square p-value**		
			
			Yoruba	Caucasian	Asian
Yoruba (n = 120)	76 (63%)	44 (37%)	-	-	-
Caucasian (n = 60)	51 (85%)	9 (15%)	0.003	-	-
Japanese/Chinese (n = 181)	163 (90%)	18 (10%)	<0.001	0.344	-
Spanish (n = 336)	294 (87.5%)	42 (12.5%)	<0.001	0.538	0.472

*For the analysis of Caucasian and Yoruba samples only the parents of the trios are considered.
