# Supplementary material for: Inter-population variability of DEFA3 gene absence: correlation with haplotype structure and population variability
Source: BMC Genomics. 2007 Jan 10;8:14. doi: 10.1186/1471-2164-8-14 (PMC1779775; doi:10.1186/1471-2164-8-14)
Supplement: Additional File 1 — Results of the association study for the three population groups. The p-values under all inheritance modes tested are shown. [file 1471-2164-8-14-S1.pdf]

SUPL TABLE 1. Association results under all inheritance modes tested for the three population groups
<NA>; not available

CEPH

	SNP	codominant	dominant	recessive	overdominant	additive
1	rs13257112	Monomorphic				
2	rs11989361	0.5668	<NA>	<NA>	<NA>	0.5668
3	rs6996047	0.9567	0.9682	0.7791	0.8829	0.8776
4	rs11996293	Monomorphic				
5	rs11996346	0.5653	0.5784	0.3037	0.8646	0.4404
6	rs4543566	Monomorphic				
7	rs13267882	0.5668	<NA>	<NA>	<NA>	0.5668
8	rs13278672	Monomorphic				
9	rs3739385	Monomorphic				
10	rs17078435	Monomorphic				
11	rs2978945	Monomorphic				
12	rs17078436	Monomorphic				
13	rs10087798	Monomorphic				
14	rs10106288	Monomorphic				
15	rs2978916	0.0045	0.001	0.1508	0.0059	0.001
16	rs2615766	Monomorphic				
17	rs2927342	Monomorphic				
18	rs12682063	0.407	<NA>	<NA>	<NA>	0.407
19	rs12682076	Monomorphic				
20	rs12675298	0.4115	<NA>	<NA>	<NA>	0.4115
21	rs2978914	Monomorphic				
22	rs10095450	0.4281	<NA>	<NA>	<NA>	0.4281
23	rs2927351	3,00E-04	<NA>	<NA>	<NA>	3,00E-04
24	rs2977814	0.0136	0.0034	0.4158	0.0059	0.0034
25	rs5028372	0.0037	0.3347	8,00E-04	0.1563	0.0085
26	rs11137085	0.0991	0.0616	0.1745	0.2292	0.0381
27	rs4841815	0.9053	<NA>	<NA>	<NA>	0.9053
28	rs4284061	0.2779	0.2981	0.1468	0.7035	0.1811
29	rs11781199	Monomorphic				
30	rs11781205	Monomorphic				
31	rs17466573	2,00E-04	0.035	0,035	0.6524	6,00E-04
32	rs7825750	0.2907	0.2223	0.1918	0.4973	0.154
33	rs6983783	Monomorphic				
34	rs4300027	0.1365	0.0468	0.5226	0.1244	0.0794
35	rs4512398	0.1365	0.0468	0.5226	0.1244	0.0794
36	rs10109569	Monomorphic				
37	rs7826487	0.3142	<NA>	<NA>	<NA>	0.3142
38	rs17078510	0.4435	<NA>	<NA>	<NA>	0.4435
39	rs7841223	0.4435	<NA>	<NA>	<NA>	0.4435
40	rs7012647	Monomorphic				
41	rs4288398	0.1498	0.126	0.1188	0.4405	0.0724
42	rs4313182	0.1188	<NA>	<NA>	<NA>	0.1188
43	rs883182	0.4073	0.971	0.1918	0.5625	0.6859
44	rs11783110	Monomorphic				
45	rs4314670	Monomorphic				
46	rs4332159	0.1188	<NA>	<NA>	<NA>	0.1188
47	rs4448289	0.4158	<NA>	<NA>	<NA>	0.4158
48	rs4469481	0.2892	0.1152	0.5632	0.1468	0.1152
49	rs9774483	0.1188	<NA>	<NA>	<NA>	0.1188
50	rs17078531	Monomorphic				
51	rs17382179	0.0039	0.0128	0.0047	0.2096	0.0019
52	rs4840665	Monomorphic				
53	rs6984215	0.1276	0.1358	0.0937	0.5625	0.0665
54	rs6605579	0.1861	0.2501	0.0873	0.8915	0.0904
55	rs7820625	0.212	0.4032	0.0905	0.9312	0.187
56	rs7816622	0.1963	0.3368	0.0905	0.9803	0.1543
57	rs7821152	Monomorphic				
58	rs4403430	0.3482	0.7168	0.1468	0.7587	0.4184
59	rs11137086	0.1188	<NA>	<NA>	<NA>	0.1188

60	rs6982814	0.1188	<NA>	<NA>	<NA>	0.1188
61	rs11776120	0.4032	0.6762	0.2409	0.3875	0.9841
62	rs6993492	0.1188	<NA>	<NA>	<NA>	0.1188
63	rs7824527	0.1199	<NA>	<NA>	<NA>	0.1199
64	rs13275347	0.5668	<NA>	<NA>	<NA>	0.5668
65	rs7825124	0.1188	<NA>	<NA>	<NA>	0.1188
66	rs4841816	Monomorphic				
67	rs4433170	0.3939	0.6381	0.2453	0.3628	0.9766
68	rs4300028	0.0463	0.0481	0.0558	0.4525	0.0192
69	rs4500099	8,00E-04	0.0018	0.0047	0.0609	3,00E-04
70	rs4576447	0.0132	<NA>	<NA>	<NA>	0.0132
71	rs6994783	Monomorphic				
72	rs7015200	0.0184	0.0165	0.035	0.2103	0.0048
73	rs17466706	8,00E-04	0.0018	0.0047	0.0609	3,00E-04
74	rs11775034	0.4202	0.8846	0.1918	0.6846	0.5863
75	rs7004995	0.0166	0.0136	0.0364	0.1833	0.0042
76	rs6982904 Geno	typing 78.3\\%				
77	rs11985030	0.0389	<NA>	<NA>	<NA>	0.0389
78	rs4840666	0.0185	0.0154	0.0378	0.1973	0.0048
79	rs17078556	0.0206	0.0174	0.0393	0.2126	0.0054
80	rs4621824	0.0184	0.0165	0.035	0.2103	0.0048
81	rs4345578	Monomorphic				
82	rs4342629	0.104	0.0341	0.4336	0.0866	0.0492
83	rs4342630	Monomorphic				
84	rs4342631	Monomorphic				
85	rs4549798	0.0223	0.0223	0.035	0.2557	0.0061
86	rs4342632	Monomorphic				
87	rs13256091	0.2574	0.1833	0.2409	0.3875	0.1274
88	rs4601339	Monomorphic				
89	rs7461488	Monomorphic				
90	rs4642671	Monomorphic				
91	rs4840667	0.1188	<NA>	<NA>	<NA>	0.1188
92	rs4546682	0.1152	<NA>	<NA>	<NA>	0.1152
93	rs6981771	0.1078	0.2764	0.0437	0.8439	0.0931
94	rs6996918	0.0971	0.2222	0.0437	0.9312	0.0736
95	rs7009276	0.0915	0.2612	0.036	0.7999	0.081
96	rs4841818	0.1833	<NA>	<NA>	<NA>	0.1833
97	rs4358823	0.1468	<NA>	<NA>	<NA>	0.1468
98	rs12716641	0.1238	0.4032	0.0437	0.6762	0.1408
99	rs12716642	0.1491	0.4032	0.0558	0.7587	0.1548
100	rs13274544	0.18	0.5271	0.0654	0.6738	0.2209
101	rs13439266	Monomorphic				
102	rs12716644	Monomorphic				
103	rs11989117	0.4115	<NA>	<NA>	<NA>	0.4115
104	rs7836636	0.3769	0.1704	0.4435	0.4405	0.1765
105	rs10086568	0.363	0.2981	0.2409	0.5469	0.2132
106	rs12674716	0.8943	0.6931	0.7089	0.9574	0.6366
107	rs13252474	0.4653	0.2656	0.3811	0.7157	0.2165
108	rs12716645	0.3809	0.1702	0.4738	0.4153	0.1828
109	rs10108420	0.9034	0.7062	0.7362	0.9449	0.6522
110	rs12716647	0.1792	0.3295	0.07	0.9348	0.1518
111	rs13261710	0.6097	0.4032	0.4281	0.8439	0.3216
112	rs4304345	0.0905	<NA>	<NA>	<NA>	0.0905
113	rs7017864	Monomorphic				
114	rs7017866	0.6578	0.4254	0.4903	0.8022	0.3603
115	rs6992098	0.494	0.3402	0.3173	0.9099	0.24
116	rs13279261	0.6198	0.4491	0.3969	0.9388	0.3376
117	rs2979406	Monomorphic				
118	rs2979405	Monomorphic				
119	rs6988346	0.4472	0.2103	0.8281	0.2741	0.2939
120	rs4260915	0.3037	0.2303	0.2323	0.4756	0.1606
121	rs4260916	0.4454	0.4415	0.2475	0.7511	0.3129
122	rs4379464	0.4448	0.4405	0.2453	0.736	0.3154
123	rs12682030	0.0558	<NA>	<NA>	<NA>	0.0558

124	rs13269815	0.4565	0.2259	0.939	0.2501	0.3341
125	rs6998006	0.6097	0.4032	0.4281	0.8439	0.3216
126	rs6998687	0.5625	0.3824	0.3744	0.8846	0.2875
127	rs6991235	0.4357	0.2074	0.4835	0.5101	0.2137
128	rs6999181	0.0937	<NA>	<NA>	<NA>	0.0937
129	rs2979404	Monomorphic				
130	rs11773934	Monomorphic				
131	rs11137087	0.1401	0.2295	0.0583	0.9092	0.1027
132	rs10503360	0.5663	0.4032	0.3598	0.9312	0.2943
133	rs4446760	Monomorphic				
134	rs9644778	0.2759	0.1469	0.3384	0.2531	0.1204
135	rs10095331	0.081	0.0639	0.0842	0.4244	0.0253
136	rs10098290	Monomorphic				
137	rs4395911	0.0937	<NA>	<NA>	<NA>	0.0937
138	rs4610776	0.1177	0.0639	0.1506	0.3628	0.0393
139	rs6988319	0.138	0.0743	0.7791	0.049	0.1856
140	rs4841831	0.1419	0.4076	0.0483	0.3518	0.0847
141	rs10093453	0.4139	<NA>	<NA>	<NA>	0.4139
142	rs11994229	Monomorphic				
143	rs17078614	0.1918	<NA>	<NA>	<NA>	0.1918
144	rs10100920	Monomorphic				
145	rs7834209	0.007	0.1523	0.0018	0.3796	0.0057
146	rs12547547	Monomorphic				
147	rs4240691	0.0014	0.0806	4,00E-04	0.8846	0.0025
148	rs4448295	0.7526	<NA>	<NA>	<NA>	0.7526
149	rs17078622	Monomorphic				
150	rs17078623	0.2317	<NA>	<NA>	<NA>	0.2317
151	rs13280193	Monomorphic				

YORUBA

	SNP	codominant	dominant	recessive	overdominant	additive
1	rs2702908	0.3559	<NA>	<NA>	<NA>	0.3559
2	rs13257112	0.181	<NA>	<NA>	<NA>	0.181
3	rs17381964	Monomorphic				
4	rs11989361	0.349	0.4462	0.1828	0.6511	0.3175
5	rs2978950	0.6106	0.355	0.8771	0.3758	0.5989
6	rs6996047	0.685	<NA>	<NA>	<NA>	0.685
7	rs11996346	0.2616	0.2649	0.1747	0.4373	0.1788
8	rs4543566	Monomorphic				
9	rs13267882	Monomorphic				
10	rs13278672	Monomorphic				
11	rs2978947	0.5435	0.6045	0.2965	0.7747	0.4865
12	rs3739385	Monomorphic				
13	rs2702875	0.0852	0.6798	0.0267	0.357	0.1698
14	rs17078435	0.7266	0.5378	0.7281	0.4317	0.7178
15	rs2951842	0.7243	0.943	0.4536	0.6563	0.8117
16	rs2978945	Monomorphic				
17	rs2978944	0.2834	0.4093	0.1331	0.7191	0.2584
18	rs2978939	0.0379	0.5472	0.0343	0.0855	0.7699
19	rs2978937	0.5285	0.5424	0.2986	0.7103	0.4338
20	rs17078436	0.1247	0.5438	0.1113	0.0529	0.5715
21	rs2738135	0.3171	<NA>	<NA>	<NA>	0.3171
22	rs2977819	0.4223	<NA>	<NA>	<NA>	0.4223
23	rs2738132	0.4935	<NA>	<NA>	<NA>	0.4935
24	rs10087798	0.6511	<NA>	<NA>	<NA>	0.6511
25	rs10106288	0.6511	<NA>	<NA>	<NA>	0.6511
26	rs2978916	0.2916	<NA>	<NA>	<NA>	0.2916
27	rs2615766	0.1862	0.0984	0.2986	0.1522	0.0787
28	rs2927342	Monomorphic				
29	rs12682063	0.8034	<NA>	<NA>	<NA>	0.8034
30	rs12682076	Monomorphic				
31	rs12675298	0.9459	<NA>	<NA>	<NA>	0.9459

32	rs2978915	0.0504	0.8247	0.0168	0.3559	0.3051
33	rs2738113	0.224	1,224	0.0966	0.3147	0.4456
34	rs2738110	0.2915	0.7926	0.1194	0.4866	0.3623
35	rs2978914	Monomorphic				
36	rs10095450	0.3551	<NA>	<NA>	<NA>	0.3551
37	rs11137081	0.853	<NA>	<NA>	<NA>	0.853
38	rs5028372 Geno	typing 78.3\\%				
39	rs11137085	0.2544	<NA>	<NA>	<NA>	0.2544
40	rs4841815	0.8946	0.6511	0.8092	0.7116	0.6371
41	rs4284061	0.5556	0.8909	0.2965	0.7116	0.9325
42	rs11781199	Monomorphic				
43	rs11781205	0.187	<NA>	<NA>	<NA>	0.187
44	rs17466573	Monomorphic				
45	rs7825750	0.734	0.9298	0.4714	0.6511	0.8323
46	rs6983783	0.5322	0.3143	0.4197	0.5775	0.2616
47	rs4300027	0.9179	<NA>	<NA>	<NA>	0.9179
48	rs4512398	0.2545	0.2523	0.1828	0.4008	0.1742
49	rs10109569	Monomorphic				
50	rs7826487	0.8261	0.6593	0.7964	0.5378	0.8497
51	rs17078510	0.4714	<NA>	<NA>	<NA>	0.4714
52	rs7841223	0.7324	0.4505	0.6645	0.6309	0.4368
53	rs7012647	Monomorphic				
54	rs4288398	0.4052	<NA>	<NA>	<NA>	0.4052
55	rs4313182	0.1848	<NA>	<NA>	<NA>	0.1848
56	rs883182	0.4858	0.4566	0.2965	0.6045	0.3648
57	rs11783110	Monomorphic				
58	rs4314670	Monomorphic				
59	rs4332159	0.2916	<NA>	<NA>	<NA>	0.2916
60	rs4448289	Monomorphic				
61	rs4469481	0.9693	<NA>	<NA>	<NA>	0.9693
62	rs9774483	0.2916	<NA>	<NA>	<NA>	0.2916
63	rs17078531	0.809	0.5342	0.7392	0.6146	0.5151
64	rs17382179	0.3121	<NA>	<NA>	<NA>	0.3121
65	rs4840665	Monomorphic				
66	rs6984215	0.659	<NA>	<NA>	<NA>	0.659
67	rs6605579	0.5173	0.357	0.3706	0.5934	0.2691
68	rs7820625	0.7249	0.7344	0.4273	0.9122	0.5384
69	rs7816622	0.5605	0.7244	0.4142	0.3402	0.8618
70	rs7821152	Monomorphic				
71	rs4403430	0.2121	0.2544	0.3582	0.1022	0.5605
72	rs11137086	0.2916	<NA>	<NA>	<NA>	0.2916
73	rs6982814	0.2916	<NA>	<NA>	<NA>	0.2916
74	rs11776120	0.2322	0.1394	0.2854	0.2118	0.1094
75	rs6993492	0.1118	0.6798	0.0578	0.2761	0.8431
76	rs7824527	0.3424	<NA>	<NA>	<NA>	0.3424
77	rs13275347	Monomorphic				
78	rs7825124	0.4735	<NA>	<NA>	<NA>	0.4735
79	rs4841816	Monomorphic				
80	rs4433170	0.4125	0.3335	0.2965	0.4566	0.2651
81	rs4300028	0.6692	<NA>	<NA>	<NA>	0.6692
82	rs4500099	Monomorphic				
83	rs4576447	Monomorphic				
84	rs6994783	0.6692	<NA>	<NA>	<NA>	0.6692
85	rs7015200	0.5921	0.7934	0.3891	0.4505	0.8647
86	rs17466706	Monomorphic				
87	rs11775034	0.1809	0.0949	0.2965	0.1469	0.0759
88	rs7004995	0.7484	0.7493	0.4482	0.9072	0.5653
89	rs6982904	0.4047	0.5089	0.1933	0.9743	0.2789
90	rs4840666	0.2976	0.4008	0.3628	0.1317	0.9013
91	rs4621824	0.2912	0.8247	0.1611	0.3335	0.6987
92	rs4345578	0.179	<NA>	<NA>	<NA>	0.179
93	rs4342629	0.8885	0.7237	0.6693	0.9819	0.6321
94	rs4342630	Monomorphic				
95	rs4342631	Monomorphic				

96	rs4549798	0.9258	0.8125	0.84	0.6968	0.9576
97	rs4342632	Monomorphic				
98	rs13256091	0.0947	0.1024	0.2965	0.0504	0.2203
99	rs4601339	Monomorphic				
100	rs4367573	0.4195	0.6006	0.3046	0.2831	0.9831
101	rs7461488	Monomorphic				
102	rs4642671	Monomorphic				
103	rs4546682	0.5213	<NA>	<NA>	<NA>	0.5213
104	rs6981771	0.4073	0.6717	0.2745	0.3035	0.8954
105	rs6996918	0.5725	0.57	0.4714	0.357	0.8589
106	rs7009276	0.4525	0.7263	0.2843	0.357	0.8634
107	rs4841818	0.8252	<NA>	<NA>	<NA>	0.8252
108	rs4358823	0.1848	<NA>	<NA>	<NA>	0.1848
109	rs12716641	0.6046	0.6309	0.4714	0.4008	0.9156
110	rs12716642	0.9356	0.7582	0.7881	0.8465	0.7211
111	rs13274544	0.8361	<NA>	<NA>	<NA>	0.8361
112	rs13439266	0.0674	<NA>	<NA>	<NA>	0.0674
113	rs12716644	Monomorphic				
114	rs11989117	0.1037	<NA>	<NA>	<NA>	0.1037
115	rs7836636	0.4647	0.2201	0.6914	0.3335	0.3083
116	rs10086568	0.9526	0.9278	0.7964	0.7934	0.9479
117	rs12674716	0.303	0.3663	0.4092	0.1223	0.936
118	rs13252474	0.5227	0.6232	0.4588	0.2631	0.904
119	rs12716645	0.3582	0.8606	0.224	0.2238	0.5807
120	rs10108420	0.5188	0.2635	0.8968	0.3821	0.4388
121	rs12716647	0.6608	0.4998	0.7776	0.3732	0.8012
122	rs13261710	0.6088	0.3807	0.4052	0.7747	0.3198
123	rs13270539	0.4345	0.784	0.2106	0.3993	0.3904
124	rs4304345	0.5604	<NA>	<NA>	<NA>	0.5604
125	rs7017585	0.4284	0.2043	0.6139	0.3017	0.2939
126	rs7017864	Monomorphic				
127	rs7017866	0.4246	0.9278	0.2487	0.2856	0.5666
128	rs6992098	0.4256	0.9554	0.224	0.3266	0.4804
129	rs13279261	0.4349	0.1969	0.5213	0.4006	0.2461
130	rs2979406	Monomorphic				
131	rs2979405	Monomorphic				
132	rs6988346	0.3137	0.1604	0.2843	0.3878	0.1279
133	rs4841822	0.6976	<NA>	<NA>	<NA>	0.6976
134	rs4260915	0.9142	0.7636	0.8434	0.6727	0.9087
135	rs4260916	0.7837	0.5278	0.9109	0.5045	0.6993
136	rs4379464	0.9526	0.9278	0.7964	0.7934	0.9479
137	rs12682030	0.5604	<NA>	<NA>	<NA>	0.5604
138	rs13269815	0.2871	0.1901	0.1676	0.6038	0.1228
139	rs6998006	0.4246	0.9278	0.2487	0.2856	0.5666
140	rs6998687	0.3389	0.1473	0.3617	0.4566	0.1591
141	rs6991235	0.3389	0.1473	0.3617	0.4566	0.1591
142	rs6999181	0.5604	<NA>	<NA>	<NA>	0.5604
143	rs2979404	Monomorphic				
144	rs11773934	Monomorphic				
145	rs11137087	0.6486	0.5267	0.692	0.357	0.883
146	rs10503360	0.2925	0.2484	0.1464	0.8908	0.1265
147	rs4446760	Monomorphic				
148	rs10867025	0.3693	0.4849	0.1599	0.8753	0.2636
149	rs9644778	0.9615	0.7917	0.9888	0.7909	0.8513
150	rs7839771	Monomorphic				
151	rs10095331	0.6859	0.8953	0.3891	0.7263	0.6289
152	rs10098290	Monomorphic				
153	rs4395911	0.983	0.853	0.937	0.8797	0.8644
154	rs4610776	0.9843	0.8953	0.937	0.8611	0.9449
155	rs6988319	0.6805	<NA>	<NA>	<NA>	0.6805
156	rs4841831	0.2024	<NA>	<NA>	<NA>	0.2024
157	rs10093453	0.7281	<NA>	<NA>	<NA>	0.7281
158	rs11994229	0.1194	0.2523	0.1376	0.1089	0.535
159	rs17078614	0.3807	<NA>	<NA>	<NA>	0.3807

160	rs10100920	0.8092	<NA>	<NA>	<NA>	0.8092
161	rs7834209	0.1006	<NA>	<NA>	<NA>	0.1006
162	rs12547547	Monomorphic				
163	rs4240691	0.8879	0.9278	0.659	0.7263	0.8896
164	rs4448295	Monomorphic				
165	rs17078622	0.8381	0.57	0.7612	0.6593	0.5529
166	rs17078623	0.6057	<NA>	<NA>	<NA>	0.6057
167	rs13280193	Monomorphic				

CHINESE-JAPANESE

	SNP	codominant	dominant	recessive	overdominant	additive
1	rs13257112	Monomorphic				
2	rs17381964	Monomorphic				
3	rs6996047	0.5485	<NA>	<NA>	<NA>	0.5485
4	rs11996293	Monomorphic				
5	rs11996346	0.5548	<NA>	<NA>	<NA>	0.5548
6	rs4543566	Monomorphic				
7	rs13267882	Monomorphic				
8	rs13278672	Monomorphic				
9	rs2978947	0.8501	0.6326	0.9659	0.5997	0.7885
10	rs3739385	Monomorphic				
11	rs2702875	0.8863	0.6688	1,6688	0.6613	0.795
12	rs17078435	Monomorphic				
13	rs2978945	Monomorphic				
14	rs2978944	0.807	0.5643	1,5643	0.5634	0.7246
15	rs17078436	Monomorphic				
16	rs2977819	0.8482	0.6639	0.9019	0.5784	0.8426
17	rs10087798	Monomorphic				
18	rs10106288	Monomorphic				
19	rs2978916	0.4651	0.6995	0.3494	0.2608	0.7981
20	rs2615766	Monomorphic				
21	rs2927342	Monomorphic				
22	rs12682063	0.5485	<NA>	<NA>	<NA>	0.5485
23	rs12682076	Monomorphic				
24	rs12675298	Monomorphic				
25	rs2978915	0.6643	0.5987	0.3744	0.8035	0.4168
26	rs2738113	0.7522	0.6995	0.4531	0.8035	0.5179
27	rs2738110	0.6643	0.5987	0.3744	0.8035	0.4168
28	rs2978914	Monomorphic				
29	rs10095450	0.0531	<NA>	<NA>	<NA>	0.0531
30	rs11137081	0.9512	<NA>	<NA>	<NA>	0.9512
31	rs5028372 Geno	typing 77.8\\%				
32	rs4841815	0.5186	0.3261	0.4051	0.475	0.2798
33	rs4284061	0.3377	0.1575	0.9189	0.1887	0.2758
34	rs11781199	0.233	<NA>	<NA>	<NA>	0.233
35	rs11781205	0.2603	<NA>	<NA>	<NA>	0.2603
36	rs17466573	Monomorphic				
37	rs7825750	0.6913	0.7278	0.4051	0.9266	0.598
38	rs6983783	Monomorphic				
39	rs4300027	0.4244	0.4177	0.2412	0.8257	0.2358
40	rs10109569	Monomorphic				
41	rs7826487	0.3949	0.4942	0.4268	0.2164	0.858
42	rs17078510	0.3949	0.4942	0.4268	0.2164	0.858
43	rs7841223	0.3949	0.4942	0.4268	0.2164	0.858
44	rs7012647	Monomorphic				
45	rs4288398	0.5943	0.4177	0.4268	0.7278	0.3134
46	rs4313182	0.8282	0.6944	0.5859	0.8004	0.6324
47	rs883182	0.6659	0.6342	0.4051	0.8257	0.5209
48	rs11783110	Monomorphic				
49	rs4314670	Monomorphic				
50	rs4332158	0.2617	0.1863	0.2051	0.4808	0.1264
51	rs4332159	0.7799	0.7852	0.5585	0.6678	0.9116
52	rs4448289	Monomorphic				

53	rs4469481	0.7939	0.8444	0.5485	0.7212	0.9693
54	rs9774483	0.7799	0.7852	0.5585	0.6678	0.9116
55	rs17078531	Monomorphic				
56	rs17382179	Monomorphic				
57	rs4840665	Monomorphic				
58	rs6984215	0.4753	0.5784	0.2412	0.9705	0.324
59	rs6605579	0.7043	0.5987	0.6223	0.4183	0.8651
60	rs7820625	0.8346	0.6701	0.6072	0.9266	0.5611
61	rs7816622	0.8227	0.9313	0.5352	0.7579	0.7021
62	rs7821152	Monomorphic				
63	rs4403430	0.7735	0.475	0.7852	0.7278	0.5554
64	rs11137086	0.7799	0.7852	0.5585	0.6678	0.9116
65	rs6982814	0.7873	0.8141	0.5536	0.6938	0.9398
66	rs11776120	0.5849	0.9705	0.3048	0.7278	0.7252
67	rs6993492	0.7873	0.8141	0.5536	0.6938	0.9398
68	rs7824527	0.7408	0.6739	0.5762	0.5666	0.8052
69	rs13275347	Monomorphic				
70	rs7825124	0.7799	0.7852	0.5585	0.6678	0.9116
71	rs4841816	Monomorphic				
72	rs4433170	0.384	0.1887	0.5585	0.2348	0.1738
73	rs4300028	0.6388	0.4942	0.4268	0.8257	0.3606
74	rs4500099	Monomorphic				
75	rs4576447	Monomorphic				
76	rs6994783	Monomorphic				
77	rs7015200	0.5518	0.5745	0.4588	0.2792	0.9159
78	rs17466706	Monomorphic				
79	rs11775034	0.5849	0.4633	0.4051	0.6342	0.3807
80	rs7004995	0.6084	0.5519	0.5519	0.3188	1,3188
81	rs4840666	0.1673	0.2725	0.3482	0.059	0.8466
82	rs17078556	0.3819	0.4127	0.4588	0.1664	0.9014
83	rs4621824	0.3059	0.4127	0.3494	0.1297	0.9774
84	rs4342629	0.2066	0.3115	0.3821	0.0762	0.8663
85	rs4342630	Monomorphic				
86	rs4342631	Monomorphic				
87	rs4549798	0.3518	0.3891	0.4399	0.1493	0.892
88	rs4342632	Monomorphic				
89	rs13256091	0.4954	0.567	0.2412	0.9839	0.348
90	rs4601339	Monomorphic				
91	rs4367573	0.7378	0.6326	0.4497	0.9659	0.4893
92	rs7461488	Monomorphic				
93	rs4642671	Monomorphic				
94	rs4546682	0.6017	0.9839	0.3344	0.4633	0.5574
95	rs6981771	0.6463	0.8596	0.3731	0.6706	0.5935
96	rs6996918	0.7021	0.567	0.4268	0.9019	0.437
97	rs7009276	0.4937	0.5027	0.2478	0.9332	0.314
98	rs4841818	0.5027	0.6639	0.2412	0.8737	0.4061
99	rs4358823	0.5027	0.6639	0.2412	0.8737	0.4061
100	rs12716641	0.7021	0.567	0.4268	0.9019	0.437
101	rs12716642	0.7021	0.567	0.4268	0.9019	0.437
102	rs13274544	0.7282	0.6156	0.4648	0.8036	0.5261
103	rs13439266	Monomorphic				
104	rs12716644	Monomorphic				
105	rs11989117	Monomorphic				
106	rs7836636	0.1258	0.249	0.0447	0.3861	0.0519
107	rs10086568	0.636	0.3951	0.5585	0.475	0.3639
108	rs12674716	0.6638	0.3719	0.7024	0.5011	0.4456
109	rs13252474	0.5262	0.2575	0.6706	0.4177	0.318
110	rs12716645	0.7623	0.4622	0.8004	0.5962	0.528
111	rs10108420	0.3363	0.2407	0.259	0.5371	0.1704
112	rs12716647	0.7623	0.4622	0.8004	0.5962	0.528
113	rs13261710	0.5262	0.2575	0.6706	0.4177	0.318
114	rs13270539	0.0619	0.0666	1,0666	0.0236	0.2142
115	rs4304345	0.2474	0.2882	0.4268	0.1088	0.619
116	rs7017864	Monomorphic				

117	rs7017866	0.473	0.2214	0.6476	0.3861	0.2806
118	rs6992098	0.5855	0.3032	0.7416	0.4277	0.3774
119	rs13279261	0.5569	0.2792	0.6476	0.461	0.33
120	rs2979406	Monomorphic				
121	rs2979405	Monomorphic				
122	rs6988346	0.4304	0.3861	0.3992	0.2608	0.6138
123	rs4841822	0.5672	0.3195	0.5927	0.3831	0.2983
124	rs4260915	0.6576	0.4179	0.5659	0.5011	0.3845
125	rs4260916	0.773	0.5748	0.5811	0.6706	0.5267
126	rs4379464	0.636	0.3951	0.5585	0.475	0.3639
127	rs12682030	0.2474	0.2882	0.4268	0.1088	0.619
128	rs13269815	0.6299	0.7278	0.4051	0.5457	0.9372
129	rs6998006	0.5262	0.2575	0.6706	0.4177	0.318
130	rs6998687	0.473	0.2214	0.6476	0.3861	0.2806
131	rs6991235	0.5262	0.2575	0.6706	0.4177	0.318
132	rs6999181	0.2474	0.2882	0.4268	0.1088	0.619
133	rs2979404	Monomorphic				
134	rs11773934	Monomorphic				
135	rs11137087	0.473	0.2214	0.6476	0.3861	0.2806
136	rs10503360	0.5933	0.3129	0.5503	0.5853	0.3276
137	rs4446760	Monomorphic				
138	rs10867025	0.669	0.9832	0.3736	0.7861	0.7864
139	rs9644778	0.8394	<NA>	<NA>	<NA>	0.8394
140	rs10095331	0.2474	0.2882	0.4268	0.1088	0.619
141	rs10098290	Monomorphic				
142	rs4395911	0.2059	0.2348	0.4268	0.0846	0.5446
143	rs4610776	0.7651	<NA>	<NA>	<NA>	0.7651
144	rs6988319	0.2474	0.538	0.0948	0.5997	0.2022
145	rs4841831	0.9032	0.7688	0.6678	0.9266	0.665
146	rs10093453	0.7179	0.6639	0.6678	0.4177	0.9708
147	rs11994229	Monomorphic				
148	rs17078614	0.7591	0.5665	0.5585	0.6706	0.5149
149	rs10100920	Monomorphic				
150	rs7834209	0.8459	0.7339	0.5745	0.8802	0.589
151	rs12547547	Monomorphic				
152	rs4240691	0.9032	0.7688	0.6678	0.9266	0.665
